# Successful Treatment of Opioid Dependence with Flexible Doses of Injectable Prolonged Release Buprenorphine

**DOI:** 10.1155/2019/9381346

**Published:** 2019-07-10

**Authors:** Oscar D'Agnone

**Affiliations:** The OAD Clinic, London, UK

## Abstract

Opioid dependence (OD) may be effectively treated with well-evidenced regimens including psychosocial and pharmacotherapeutic interventions. Treatment has benefits but also limitations including risk of diversion, impact of mandatory daily supervision, and stigma. An injectable prolonged release buprenorphine with flexible dose options has recently been approved by the European Commission and is available in the UK and other European Countries. Initial positive treatment experience in patients with different clinical scenarios (patients with no recent treatment history, a transfer from oral methadone, and change from sublingual buprenorphine tablets) provides evidence of the potential benefit in a range of situations for this therapeutic option. Adoption of the injectable form was clinically successful with no withdrawal signs, nor evidence of use of other drugs. Patient reported outcomes were positive including reduction in cravings and anxiety and improved attitude, relationships, and general mood.

## 1. Introduction

Opioid dependence (OD) is an important individual and public health issue: [1] adverse health outcomes [2] and negative social impacts include unemployment, homelessness, family disruption, lower economic productivity, social instability, crime, and economic burden [3–5]. Treatment with psychosocial and pharmacotherapeutic interventions is effective [6]. Some people do not achieve the treatment outcomes they desire.

Common medication choices include oral methadone or buprenorphine [2, 7, 8]. Oral medication has an inherent risk of diversion [9]. Dispensing with mandatory supervised consumption is common, especially at the start of therapy [7]. Daily supervision limits the ability to work and can lead to discrimination. Treatment itself may be marginalising for many people and can promote stigma. Suboptimal dosing and more common use “on top” of illicitly sourced opioids and other drugs are often observed related to challenges in adherence [10].

Prolonged release buprenorphine [11, 12] indicated for the treatment of OD achieves clinically effective, stable buprenorphine levels for weekly or monthly periods after injection [13–15].

Case narratives presented here describe the successful initiation and early period of treatment with this pharmacotherapy option in 3 different scenarios which help to define situations for recommendation of this treatment option.

## 2. Cases

### 2.1. Case Study 1: Transfer from Sublingual Buprenorphine Tablets

A 52-year-old Caucasian male with OD began treatment with prolonged release buprenorphine after inadequate progress on other treatment options.

OD history included a 22-year duration of intermittent injected heroin use, no major other medical or mental health history present. Family/relationship history: married (no history of OD in wife), two children, living with family, successfully employed in a senior management role in an information technology company.

OD developed from age of 30 years: initially he smoked up to 0.5g of heroin daily for 10 years; injected heroin was used for 2 years until treatment was first commenced with slow release morphine 300 mg twice daily at a different treatment service. Treatment engagement ceased in 2016, replaced with smoking heroin and use of illicitly sourced slow release morphine to control withdrawals.

Upon presentation the patient reported use of slow release morphine 100 mg/d in the period up to 24 hours before assessment. The patient's attitude was open and friendly; he was casually dressed with normal speech rate and volume, in a good mood, showing no evidence of self-harm or suicidal ideation with no perceptual or cognitive alterations. There were no prescribed medications from other healthcare professionals.

Upon taking a history and examination, a diagnosis of ICD-10 opioid dependence was made; urine drug screen (UDS) was positive for morphine, cocaine, and benzodiazepine. The patient reported snorting cocaine one week prior to attending the clinic and using borrowed diazepam to facilitate sleep but that these drugs were not used regularly. Weight was 70 kg, height, 178 cm; blood pressure, 133/80mmHg; pulse, regular at 62 bpm; pulse oximetry recorded an oxygen saturation of 97%. Very mild opioid withdrawal symptoms, moderate restlessness, and anxiety were present.

Upon diagnosis, an initial treatment plan including supervised consumption of sublingual buprenorphine 8 mg (2mg tablets, 4 times daily), increasing to a total daily dose of 12 mg and then 14 mg, was agreed. The patient noted the challenges of regular oral medication and work life: during the initial consultation, he requested the possibility of starting prolonged release buprenorphine therapy at a suitable time.

Five days after treatment initiation, the patient attended a standard review; he was punctual, in good mood, and with a positive attitude. Current treatment was oral buprenorphine. UDS was positive only for buprenorphine, confirming engagement and adherence with the treatment regimen. Patient reported outcomes included improved sleep and general aspect or mood, as confirmed by his wife. Observations were normal; there were no signs of opioid withdrawal.

As per treatment plan, prolonged release buprenorphine, 24 mg (product details: Buvidal®, Camurus AB, Lund, Sweden), was administered by subcutaneous injection at the right lower abdominal side below belt line. (This provides a constant plasma level of buprenorphine equivalent to 12-16mg sublingual buprenorphine for 1 week following injection.) After a period of 2 hours the patient left the clinical facility with no signs of withdrawal and in a good mood. At one-week follow-up, the patient remained stable; UDS showed positive for buprenorphine only. A further injection of prolonged release buprenorphine was administered (96mg, to provide the equivalent of daily sublingual dose 12-16mg treatment for one month).

Three months after starting therapy, the patient has abstained from all drugs, including alcohol and tobacco. He reports that cravings have been almost completely absent with improvements in all aspects of his life. His relationships with his wife, children, and friends improved significantly. The patient reports,* “My life has changed. My mood has changed. You should read the text messages my wife used to send me before and after the injection.”*

### 2.2. Case Study 2: Transfer from Methadone

A 56-year-old Caucasian female with more than 25 years' history of heroin use chose prolonged release buprenorphine treatment as a part of a care plan for OD.

History included mainly smoking heroin; she injected only twice in the 1990s and never shared injecting equipment. She received treatment for OD at a clinic in the 1990s with no use of illicit drugs for several years following discharge.

In July 2017, the patient began to use heroin and after expert consultation planned to take 10 mg of oral buprenorphine resulting in a clinically stable situation for several months. The patient endured a period of intense stress due to the illness of a family member. At this time buprenorphine therapy was stopped, related to a painful surgical intervention in her mouth treated with prescribed opioid painkillers.

Prior to the most recent consultation, the patient reported smoking heroin up to 0.6g/d and occasional smoking of cannabis and with no other illicit drug use. Medical history included obstructive pulmonary disease and moderate depression for which mirtazapine was prescribed since 2017. The patient reported feeling down; there were no reports of self-harm nor suicidal ideation nor signs of disordered thought or perception. The patient lives alone in her house but visits her own children and grandchildren; daily activities are centred on caring for her family member who is in poor health. The patient has an extensive network of friends including persons with no history of OD.

On assessment the patient was casually dressed, engaged with normal eye contact and normal speech rate, tone, and volume. The patient reported she was anxious, felt guilty following the recent relapse, and “wanted to stop using heroin as soon as possible.” Observations were normal: blood pressure 122/86mmHg and pulse regular at 87 bpm; pulse oximetry recorded an oxygen saturation of 95%; weight was 59 kg; height was 163 cm. UDS was positive for tetrahydrocannabinol and morphine.

Initially a treatment plan based on oral methadone was agreed; a dose of 60 ml per day was achieved. The patient started to smoke heroin daily in addition to the prescribed methadone. Treatment plan was changed with an interim reduction in methadone to facilitate a change to oral buprenorphine for a week prior to starting treatment with prolonged release buprenorphine based on an equivalent dose to 16 mg of sublingual buprenorphine. The new treatment choice was commenced with weekly injections with the aim to progress to monthly dosing if possible.

On recent review, the patient was receiving a monthly injection of 96 mg prolonged release buprenorphine, Buvidal®, equivalent to 12-16mg daily oral therapy. Patient reported outcomes were positive including feeling stable and happy because she was able to abstain from heroin use “*for the first time in ages.*”

There were no signs of opioid withdrawal and UDS was negative for morphine. The patient has continued treatment for 4 months, remaining stable with urine tests positive only for buprenorphine.

### 2.3. Case Study 3: No Recent Treatment History

The patient is a 48-year-old male, employed as a skilled tradesman in a family business who considered prolonged release buprenorphine treatment.

He reported a 26-year history of opioid dependence including use of injected heroin. Past engagement with treatment included a buprenorphine-led detoxification program and naltrexone implant. The patient was abstinent for a period of 6 months, followed by relapse in 2005. Since his relapse, the patient has not engaged with any treatment for OD.

He presented for detoxification treatment in early 2019. He attended the appointment punctually with an open attitude. He was casually dressed and maintained normal eye contact with normal speech rate, tone, and volume. He was in a good mood and showed no signs of disordered thought or perception. He has no history of mental health problems and denies any current self-harm or suicidal ideation. He maintains a good relationship with his family (including a child) while living alone in his own house. Current use of drugs was reported to include injected use of heroin three to four times per day; estimated at 0.8g/d, smoking one small rock of crack cocaine weekly, 10 cigarettes daily, and no other illicit drug or alcohol use.

On initial assessment at presentation, observations were unremarkable. Blood pressure was 136/75mmHg, pulse rate was regular at 63 bpm, pulse oximetry was normal SpO2 98%, weight was 80 kg, and height was 177cm. UDS was positive for morphine, methadone, and cocaine. A treatment plan was agreed and commenced on oral buprenorphine-naloxone. On the second day of treatment at review the patient reported feeling better in general; discussion included appropriate dosing and future plans. The option of a weekly or monthly injection of buprenorphine was considered and agreed on.

The patient attended two days later to commence prolonged release buprenorphine therapy. The current dose of sublingual buprenorphine at this time was 16 mg daily. He was punctual, in good mood, and with a positive attitude towards the new intervention proposed. Current medication was sublingual buprenorphine-naloxone 16 mg taken under supervision at midday. Observations were normal; there were no signs of opioid withdrawal. A dose of 24 mg prolonged release buprenorphine was administered by subcutaneous injection on the left lower abdominal side below belt line providing one week of buprenorphine at levels equivalent to 12-16mg of oral medication. There were no signs of side effects in the immediate period following administration. UDS was positive for cocaine, cannabis, and morphine.

At review after one week of therapy, the patient reported he was feeling well, although concerned about potential withdrawal symptoms at the end of the week of therapy. It was agreed to continue with an injection providing treatment for a period of one month. An injection of prolonged release buprenorphine, Buvidal®, 96 mg, to provide treatment for 1 month at an equivalent to a daily oral dose of 12-16mg was administered to the right abdominal region. UDS was negative for morphine but remained positive for cocaine and tetrahydrocannabinol. The patient reported improvement in mood and was pleased to be able to apply himself full time at work, avoiding the stigma and time lost related to daily pharmacy attendance for observed consumption. His family reported he was well and were supportive of his treatment plan.

After three months, the patient continues on monthly injections of the prolonged release buprenorphine therapy; he remains clinically stable with no evidence of withdrawal. UDS does not show evidence of morphine. Occasional use of cocaine and cannabis may continue. After 3 months of continuing treatment the patient remains stable and continues to make progress on the recovery journey.

## 3. Discussion

Pharmacotherapy with oral buprenorphine or methadone for OD is effective but has limitations. Many patients are not able to maintain normal living and engage with treatment consistently, especially if daily medication pick-up and supervised consumption is required. There are particular risks of poor adherence, drop-outs from treatment, and relapse.

These case studies provide initial evidence of the benefits of prolonged release buprenorphine. Here the introduction of the product was well-tolerated and not associated with signs of withdrawal. Assessments indicated that these patients did not use other opioids during the treatment period. Patient reported outcomes were positive and similar benefits were noted by families. The impact on activities of daily life such as work is well-evidenced by these examples: these patients reported that it was easier to work avoiding stigma. These three case reports represent important and relevant clinical scenarios. It is of note that patients had a history of inadequate progress on other forms of pharmacotherapy but were able to achieve clinical stability on transfer to the injectable product. It is also of interest that improvements in mood and attitude were common with the ability to work enhanced. Such gains are expected with appropriate dosing of medication—the injectable form increases the chance of achieving this goal. These patients had social networks able to provide support to some extent; other patients may need more intensive contact with services able to provide such support and monitoring. The need or otherwise for psychosocial interventions should be decided based on needs of each individual in the context of effective pharmacotherapy. Patients with a treatment history including higher doses of methadone will require reduction on dose and transition strategies prior to any attempt to introduce the prolonged release buprenorphine product. This early experience of prolonged release buprenorphine provides evidence of success and foundation for continuing use.
